# Antimicrobial resistance and virulence profiling of *Staphylococcus pseudintermedius* isolated from cats, Bangladesh

**DOI:** 10.1080/01652176.2024.2326848

**Published:** 2024-03-15

**Authors:** Eaftekhar Ahmed Rana, Tanvir Ahmad Nizami, Md. Sayedul Islam, Subrata Sarker, Hafizar Rahman, Azizul Hoque, Mizanur Rahman

**Affiliations:** a Department of Microbiology and Veterinary Public Health, Chattogram Veterinary and Animal Sciences University, Khulshi, Chattogram, Bangladesh; b Department of Microbiology and Public Health, Bangabandhu Sheikh Mujibur Rahman Agricultural University, Gazipur, Bangladesh; c Department of Livestock Services, Ministry of Fisheries and Livestock, Dhaka, Bangladesh; d Department of Pathology and Parasitology, Chattogram Veterinary and Animal Sciences University, Khulshi, Chattogram, Bangladesh; e Teaching & Training Pet Hospital and Research Center, Chattogram Veterinary and Animal Sciences University, Khulshi, Chattogram, Bangladesh

**Keywords:** Cats, MRSP, resistance genes, *S. pseudintermedius*, virulence genes

## Abstract

*Staphylococcus pseudintermedius* is a significant bacterial pathogen that frequently colonizes different body sites and mucous membranes of pets. The objectives of the cross-sectional study were to estimate the prevalence, antimicrobial resistance pattern, and detection of diverse resistance as well as virulence genes of *S. pseudintermedius* in cats. A standard bacteriological method, species-specific gene and different antimicrobial resistance as well as virulence genes were confirmed by PCR assay. A total of 233 swab samples were collected from different body sites of 102 cats, among them 146 swabs from 73 healthy cats, and 87 from 29 diseased cats. Overall, prevalence of *S. pseudintermedius* in cats was 12.01%, while dermatitis and otitis affected cats were 26.08% and 33.33%, respectively. The highest antimicrobial resistance was observed against penicillin (96.42%) followed by streptomycin (85.71%) and erythromycin (78.57%). Moreover, 89.28% of *S. pseudintermedius* isolates exhibit multi-drug resistance (MDR) (≥ 3 classes’ antimicrobial resistant). In addition, 17.86% isolates harbored the *mec*A gene; thus, were classified as methicillin-resistant *S. pseudintermedius* (MRSP). Furthermore, the erythromycin resistance genes *erm*A and *erm*B were harbored by 25% and 10.71% of isolates, while 42.86% and 17.86% of isolates carried *tet*K and *tet*L (tetracycline resistance) genes, respectively. In virulence profiling, 32.14% (*sea*) and 10.71% (*seb*) of isolates were found positive for enterotoxin genes, whereas, the toxic shock syndrome toxin-1 (*tst-*1) gene and the Panton-Valentine leukocidin gene (*pvl*) were detected in 25% and 14.29% of isolates, respectively. To our knowledge, this is the first report of cats in Bangladesh for MDR *S. pseudintermedius*, MRSP, and their virulence profiling.

## Introduction

*Staphylococcus pseudintermedius* is a coagulase-­positive significant opportunistic pathogen that is associated with a wide variety of clinical diseases, predominantly in pet animals like dogs and cats. This organism is usually harbored in the skin and mucosa of cats and distributed in different body locations, mainly in the nares, mouth, pharynx, groin, and perineum regions (Viegas et al. 2022). This pathogen is mainly involved in skin and ear infections, namely dermatitis, pyoderma, and otitis, and sometimes leads to life-threatening septicemia (Bierowiec et al. 2021). The pathogenicity of *S. pseudintermedius* is exerted through several virulence factors and few of them are closely identical to those of *Staphylococcus aureus*. The majority of the isolates are able to produce various enzymes and toxins namely coagulase (*coa*), protease (*pst*), thermonuclease (*nuc*), exfoliative toxins (*SIET*), haemolysins (*hlb*), immunoglobulin-binding protein (*pSbi*), toxic shock syndrome toxin 1 (*tst*-1), and enterotoxins (*SEs*) (Glajzner et al. 2023). These factors are involved in various forms of pathogenesis in host body such as the conversion of fibrinogen to fibrin, hydrolysis of protein, hemolysis, cytopathic effects on epithelial cells, intoxication, etc. (Bhooshan et al. 2020). In addition, it produces leucotoxin *Luk*-I and Panton-Valentine leucocidin (*pvl*), which are highly leucotoxic to a variety of polymorphonuclear cells and cause tissue damage and necrosis (AnandaChitra et al. 2018). Moreover, the biofilms producing ­*S. pseudintermedius* have capacity to prevent the host defense mechanisms, and responsible for the establishment of chronic infection and poor drug activity on the infection site (Meroni et al. 2019). Hence, most of the virulence factors play a crucial role in every phase of infection, including bacterial colonization, transmission and generation of clinical conditions (Meroni et al. 2019; Bhooshan et al. 2020). However, to treat the clinical infection of diseased cats, the veterinarian and cat owners constantly use wide classes of antimicrobials, which ultimately creates selective pressure upon microorganisms. Consequently, *S. pseudintermedius* isolates acquire different extrinsic and intrinsic antimicrobial resistance mechanisms and exhibit resistance to diverse antimicrobials approved for use in veterinary medicine (Wang et al. 2022). *S*. *pseudintermedius* has become more significant in recent years because of the rise in methicillin and non-β-lactam antibiotic resistance (Morais et al. 2023). Recently, *mec*A gene mediated MRSP has become a significant concern in veterinary medicine for clinical management of animal health. Moreover, methicillin resistance is conferred by the encoding of *mec*A and *mec*C genes in bacterial chromosome, which codes for the synthesis of a modified penicillin-binding protein (PBP2a also called PBP2). Since β-lactam antibiotics typically bind to staphylococcal PBP to inhibit the bacterium by interfering the cell wall synthesis and these antimicrobials have a poor affinity to the modified PBP of MRSP (Krapf et al. 2019). The staphylococcal species including *S. pseudintermedius* can also acquire multidrug resistance via the acquisition of different resistance genes likely, *mec*A and *bla*Z for β-lactams, *tet*K, *tet*L and *tet*M for tetracyclines, *erm*A, *erm*B and *ermC* for erythromycin, etc. (Meroni et al. 2019).

The invasion of *S. pseudintermedius* is not only significant for disease in cats but also poses a potential threat to public health since cats can act as the reservoir of MRSP and can potentially transmit to humans (Pomba et al. 2017). Although MRSA and MRSP were described in one study regarding dogs (Rana et al. 2022a), to date no information is available about the extent of *S. pseudintermedius* in cats of Bangladesh. Therefore, the aims of the current study were to determine the prevalence of *S. pseudintermedius* in cats, their antimicrobial resistance pattern, and the detection of multiple resistance and virulence genes.

## Materials and methods

### Ethical approval and pet owner consent

All procedures were followed in compliance with the standards established by the Ethics Committee of Chattogram Veterinary and Animal Sciences University (CVASU); with the approval number CVASU/Dir (R&E) EC/2018/39 (2/8). Moreover, pet owner consent was taken before collecting the cat samples and noticed regarding the research.

### Collection and preparation of samples

All types of cats registered to a Sahedul Alam Quaderi Teaching Veterinary Hospital (SAQ -TVH), CVASU, in different time intervals from January 2018 to May 2023 (sampling was stopped for few years due to the COVID-19 pandemic) for a variety of services like veterinary care, immunization, or general health check-ups were selected for sampling. Swab samples were obtained from the oral mucosa, the surface of the perineal region for all cats. In addition, any cats with dermatitis or otitis were considered as a clinical infection or sick cats and also selected for sampling. After that, sampling was performed using a separate sterile cotton swab and placed immediately in individual 5 ml Mueller Hinton broth (MHB) (Oxoid Ltd., Basingstoke, UK) supplemented with 6.5% NaCl. Furthermore, all the samples were stored at 4 °C and sent to the Department of Microbiology and Veterinary Public Health, CVASU for further processing.

### Isolation and identification of S. pseudintermedius

**Figure 1.**
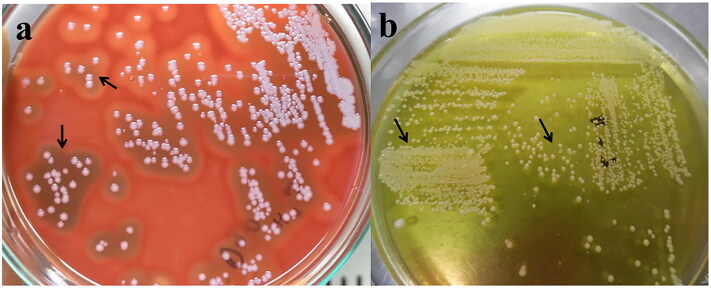
The image depicts the pure isolates of *S. pseudintermedius*; the blood agar (1a) illustrates the phenotypic appearance (round, smooth, non-pigmented or grey-white) and different hemolytic properties (alpha, beta, and double hemolysis). In addition, mannitol salt agar (1b) demonstrates the fermentation of mannitol sugar and characteristic changes of media color.

Initially, all swab samples were incubated overnight at 37 °C for primary selective enrichment. Then, 20 μL of samples from the broth cultures were plated on 5% bovine blood agar and incubated at 37 °C for 24 h. Colonies with a characteristic appearance on blood agar for staphylococci were smooth, medium-sized, raised, grey-white, hemolytic colonies (Figure 1a). The suspected colonies were further sub-cultured onto Mannitol salt agar (MSA) (Oxoid Ltd., Basingstoke, UK) and incubated for 24 h at 37 °C to observe the mannitol salt fermentation (Figure 1b), (Rana et al. 2022a). Selected presumptive colonies were again cultured into 5% bovine blood agar and confirmed the hemolytic activity, and subsequently examined by Gram’s staining and catalase tests. Finally, tube coagulase test was performed to separate the coagulase positive and negative staphylococci (Rakotovao-Ravahatra et al. 2019).

### Extraction of bacterial nucleic acid

Bacterial genomic DNA was extracted from the freshly grown coagulase positive cultures on blood agar plates using the boiling lysis method (Amin et al. 2020). In brief, 3 to 5 uniform pure isolated colonies were added in 200 µL nuclease free water within a sterile eppendorf tube and colonies were mixed by vortex machine. Then, all tubes were boiled at 100 °C for 10 min and immediately cooled at −20 °C for 5 min. After that, all the tubes were centrifuged at 15000 rpm for 5 min and finally 50 µL of supernatant was collected. The extracted DNA was preserved at −20 °C until use.

### PCR confirmation of S. pseudintermedius

Individual, DNA samples were subjected to PCR assay using the *S. pseudintermedius* specific ‘*pse’* gene described by Sasaki et al. (2010) (Table 1). A total of 50 µL of PCR reaction mixture was thermally cycled once for denaturation at 95 °C for 2 min, followed by 30 times at 95 °C for 30 s, 56 °C for 35 s, 72 °C for 1 min and then once for final extension at 72 °C for 2 min. The amplicons were measured using a 1 kb plus DNA ladder on a 1.5% agarose gel supplemented with 0.5 µg/mL of ethidium bromide, followed by visualization using an ultraviolet chamber. Previously confirmed *S. pseudintermedius* and *S. aureus* (Rana et al. 2020a) were used as a positive and negative control for monitoring the PCR reaction, respectively. All PCR-positive *S. pseudintermedius* were grown in 5 mL brain heart infusion broth (Oxoid Ltd., Basingstoke, UK) and preserved at −80 °C in CVASU microbial biobank for further analysis.

**Table 1.** Primers sequence used for the identification of species, resistance, and virulence genes amplification of *S. pseudintermedius.*

Types	Target genes	Primer sequence (5′ to 3′)	Annealing temperature	Amplicon size (bp)	Reference
Species identification	*pse*	SF: TRGGCAGTAGGATTCGTTAA SR: CTTTTGTGCTYCMTTTTGG	56 °C	926	Sasaki et al. 2010
Resistant genes	*mec*A	SF: TCCAGATTACAACTTCACCAGG SR: CCACTTCATATCTTGTAACG	59 °C	162	Larsen et al. 2008
	*erm*A	SF: TATCTTATCGTTGAGAAGGGATT SR: CTACACTTGGCTGATGAAA	55 °C	139	Shekarabi et al. 2017
	*erm*B	SF: CTATCTGATTGTTGAAGAAGCATT SR: GTTTACTCTTGGTTTAGGATCAAA	55 °C	141	
	*erm*C	SF: AATCGTCAATTCCTGCATGT SR: TAATCGTGGAATACGGGTTTG	55 °C	299	
	*tet*K	SF: TTAGGTGAAGGGTTAGGTCC SR: GCAAACTCATTCCAGAAGCA	55 °C	697	Gómez-Sanz et al. 2010
	*tet*L	SF: CATTTGGTCTTATTGGATCG SR: ATTACACTTCCGATTTCGG	55 °C	456	
	*tet*M	SF: GTTAAATAGTGTTCTTGGAG SR: CTAAGATATGGCTCTAACAA	55 °C	576	
Virulence genes	*sea*	SF: GGTTATCAATGTGCGGGTGG SR: CGGCACTTTTTTCTCTTCGG	50 °C	102	Zhou et al. 2017
	*seb*	SF: GTATGGTGGTGTAACTGAGC SR: CCAAATAGTGACGAGTTAGG	50 °C	164	
	*sec*	SF: AGATGAAGTAGTTGATGTGTATGG SR: CACACTTTTAGAATCAACCG	50 °C	451	
	*eta*	SF: ATATCAACGTGAGGGCTCTAGTAC SR: ATGCAGTCAGCTTCTTACTGCTA	50 °C	1155	
	*etb*	SF: CACACATTACGGATAATGCAAG SR: TCAACCGAATAGAGTGAACTTATCT	50 °C	604	
	*pvl*	SF: GTGCCAGACAATGAATTACCC SR: TTCATGAGTTTTCCAGTTCACTTC	55 °C	255	
	*tst*	SF: ACCCCTGTTCCCTTATCATC SR: TTTTCAGTATTTGTAACGCC	50 °C	326	

### Antimicrobial susceptibility testing of S. pseudintermedius

All PCR-confirmed S. *pseudintermedius* were ­subjected to antimicrobial sensitivity testing using the agar disk diffusion method against 12 antimicrobials of seven different classes. Inoculation was carried out using cell suspensions of 0.5 McFarland turbidity standard and results were read within 18 to 24 h according to the Clinical and Laboratory Standards Institute (CLSI) guidelines for veterinary pathogens (CLSI, 2018). The following antimicrobials (Oxoid Ltd., Basingstoke, UK) were tested: amoxicillin & clavulanic acid (30 μg), cefoxitin (10 μg), ceftriaxone (30 μg), ciprofloxacin (10 μg), gentamicin (30 μg), erythromycin (15 μg), imipenem (10 μg), oxacillin (5 μg), streptomycin (100 μg), penicillin (10 IU), sulfamethoxazole-trimethoprim (1.25 + 23.75 μg), tetracycline (30 μg). Finally, the results were interpreted as susceptible (S), intermediate (I) and resistant (R) by measuring the zone of inhibition around each disk (CLSI, 2018). Methicillin resistance was determined by evaluating the zone diameter surrounding oxacillin and cefoxitin disks (Rana et al. 2022a). Isolates of *S. pseudintermedius* that exhibited resistance against ≥3 antimicrobial classes were defined as multidrug-resistant (MDR) (Burke and Santoro 2023).

### Detection of *mec*A, erythromycin, and tetracycline resistant genes

To confirm the methicillin resistance, PCR assay was used to detect the presence of the *mec*A gene in all oxacillin and cefoxitin-resistant *S. pseudintermedius* isolates, as described by Larsen et al. (2008). In addition, all primary erythromycin and tetracycline phenotypic resistant isolates were screened by targeting specific resistant genes. Where, the *erm*A, *erm*B, and *erm*C genes (Shekarabi et al. 2017) targeted for erythromycin resistance and *tet*K, *tet*L, and *tet*M genes (Gómez-Sanz et al. 2010) for tetracycline resistance (Table 1). Previously confirmed tetracycline resistant *S. aureus* (Rana et al. 2022b) was used as positive control, while nuclease free water was used as negative control for every reaction.

### Detection of virulence genes

All PCR confirmed *S. pseudintermedius* isolates were further screened for the detection of enterotoxin genes (*sea*, *seb*, *sec*), panton-valentine leucocidin gene (*pvl)*; exfoliative toxin genes (*eta*, *etb*), and a toxic shock syndrome toxin-1 gene (*tst*-1) described by Zhou et al. 2017. The thermal cycling program comprises an initial denaturation at 94 °C for 5 min, annealing followed by 30 cycles of 94 °C for 1 min, 50 °C for 1 min (55 °C for *pvl*), and 72 °C for 1 min, with a final elongation of 72 °C for 10 min. The primers and annealing temperatures are listed in Table 1.

### Statistical analysis

Any cat was considered positive for *S. pseudintermedius* when samples from at least one of the different body sites tested positive growth of the particular organism. Calculating the prevalence involved dividing the total number of positive cats (the numerator) by the total number of cats sampled (the denominator). In addition, percentages of resistant and virulence genes were enumerated by the number of positive isolates by the total number of isolates tested. Finally, the heat map and the bar diagram were constructed using Graph pad prism (Version: 8.0).

## Results

### Samples

A total of 233 swab samples were collected from 102 cats. Among them, 146 swabs were from 73 healthy cats, and 87 were from 29 clinically sick cats. Of the total samples, 204 samples collected from the oral (102) and perineal region (102), and the remaining 23 samples and 6 samples came from clinical cases of dermatitis and otitis, respectively (Table 2**)**.

**Table 2.** Distribution of *S. pseudintermedius* and methicillin-resistant *S. pseudintermedius* (MRSP) in different body sites of clinically healthy and diseased cats.

Body sites of cat	No. swab samples	No. *S. pseudintermedius* (%, 95% CI)	No. MRSP (%, 95%CI)
Perineal	102	11 (10.78, 5.97 to 18.44)	2 (18.18, 3.99 to 48.85)
Oral	102	9 (8.82, 4.52 to 16.11)	0
Dermatitis	23	6 (26.08, 12.26 to 46.76)	3 (50.0, 18.76 to 81.24)
Otitis	6	2 (33.33, 9.25 to 70.43)	0
Total	233	28 (12.01, 8.40 to 16.86)	5 (17.86, 7.41 to 36.06)

MRSP = methicillin-resistant *S. pseudintermedius*; CI = confidence interval.

### Distribution of *S. pseudintermedius*

**Figure 2.**
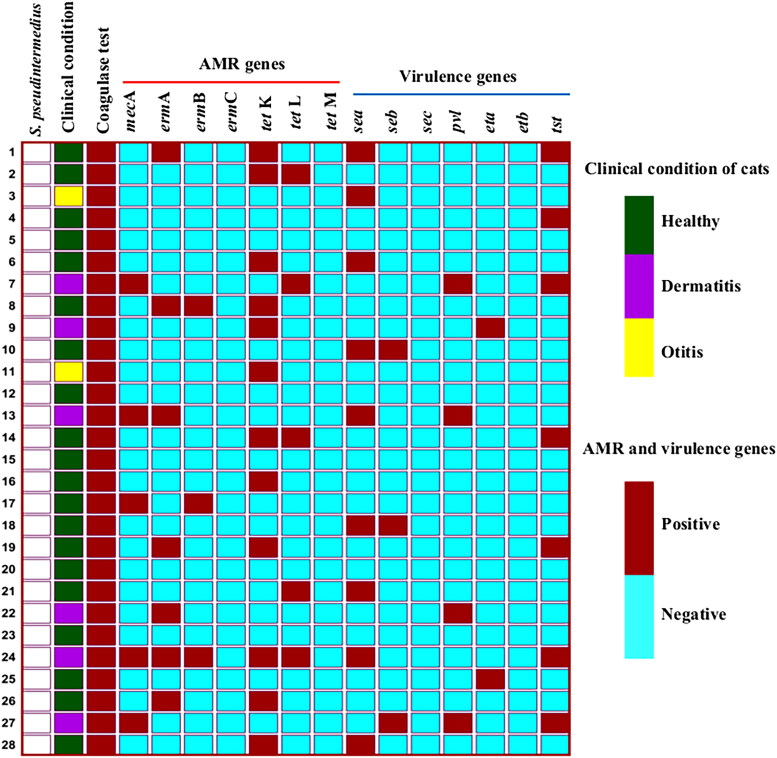
The heat map represents the clinical condition and biochemical test (coagulase) profile of *S. pseudintermedius* positive cats. In addition, it also illustrates the diverse antimicrobial resistance (AMR) and virulence genes profiles of individual isolate.

In total, 28 (12.01%; 95% CI: 8.40-16.86) distinct *S. pseudintermedius* were isolated. The highest number of *S. pseudintermedius* isolates were found in ­perineal swab (11; 10.78%; 95% CI: 5.97-18.44) ­followed by oral (9; 8.82%; 95% CI: 4.52-16.11), ­dermatitis (6; 26.08%; 95% CI: 12.26-46.76), and otitis (2; 33.33%; 95% CI: 9.25-70.43) (Figure 2).

### Antimicrobial susceptibility profiles

In antimicrobial susceptibility testing, the highest resistance was observed against penicillin (96.42%) followed by streptomycin (85.71%) and erythromycin (78.57%). Resistance against amoxicillin/clavulanic acid and ceftriaxone was detected in 60.71% and 64.28% of isolates, respectively. In addition, 57.7% of isolates showed resistance against oxacillin, whereas ciprofloxacin and cefoxitin showed a moderate level (32.14%) of resistance. However, high rates of susceptibility were observed toward gentamicin (92.86%), imipenem (89.28%), and trimethoprim/sulfamethoxazole (78.57%) against *S. pseudintermedius* (Table 3).

**Table 3.** Antimicrobial susceptibility profile of *S. pseudintermedius* (n = 28) in cats.

AST	AMC	CRO	CIP	CN	E	FOX	IPM	OX	P	SXT	S	TE
Susceptible isolates	11	10	19	26	6	19	25	12	1	22	4	8
Sensitive (%)	39.29	35.71	67.86	92.86	21.42	67.86	89.28	42.86	3.57	78.57	14.28	28.57
Resistant isolates	17	18	9	2	22	9	3	16	27	6	24	20
Resistance (%)	60.71	64.28	32.14	7.14	78.57	32.14	10.71	57.14	96.42	21.42	85.71	71.42

AST = Antimicrobial susceptibility test, AMC = amoxicillin/clavulanic acid, CRO = ceftriaxone, CIP = ciprofloxacin, CN = gentamicin, E = erythromicin, FOX = cefoxitin, IPM = imipenem, OX = oxacillin, P = penicillin, SXT = trimethoprim/sulfamethoxazole, S = streptomycin and TE = tetracycline. Where, all intermediate resistant isolates were considered as resistant.

### MDR detection rate

**Figure 3.**
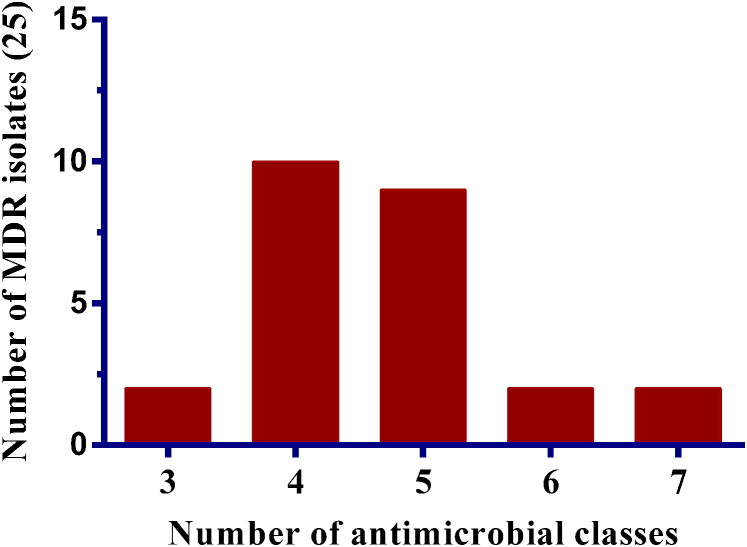
Multidrug-resistance profile of *S. pseudintermedius* isolates (n = 25), where each bar indicates the number of isolates resistant to a number of antimicrobial classes.

Among the collected isolates, 25 (89.28%) were resistant to more than three antimicrobial classes, thus they were classified as MDR. Moreover, 10 *S. pseudintermedius* isolates were found resistant to four distinct classes of antimicrobials, whereas 9 isolates were resistant to five classes. Surprisingly, two isolates were resistant to seven different antimicrobial classes (Figure 3). While individual 2 isolates (total 4 isolates) were exhibit the resistance against three and six distinct classes of antimicrobial, respectively (Figure 3).

### Frequency of antimicrobial resistant genes

**Figure 4.**
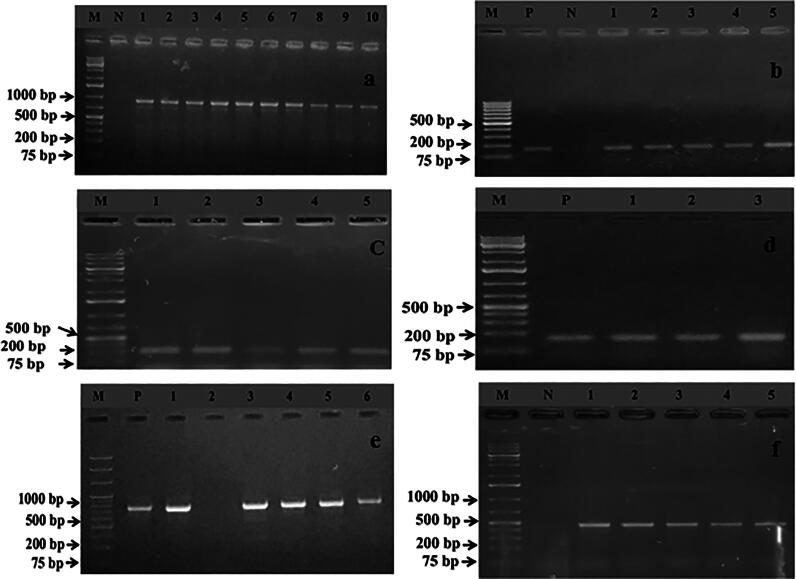
Gel electrophoresis image (4a) of PCR products of *S. pseudintermedius* (lane 1-10) showing specific amplified bands (926 bp) and image 4b (lane 1-5) exhibit the amplified *mec*A gene (162 bp) on 1.5% agarose gel. In addition, images 4c and 4d represent the amplification of the erythromycin resistance gene, namely *erm*A (139 bp) and *erm*B (141 bp), while images 4e and 4f illustrate the specific bands of the tetracycline resistance gene, like *tet*K (697 bp) and *tet*L (456 bp). Moreover, in all gel electrophoresis systems used a 1 kb plus DNA marker for confirmation of the amplification of a specific gene. Where, lane M, N, and P represent the DNA markers, negative and positive control, respectively.

Carriage of resistance genes were screened based on phenotypic resistant results of *S. pseudintermedius* (Figures 2 and 4). Within 28 isolates, 5 (17.86%) *S. pseudintermedius* harbored the *mec*A gene and designated them as MRSP. Resistance to erythromycin was confirmed by the detection of *erm*A and *erm*B genes, which were 7 (25%) and 3 (10.71%), respectively (Figure 4). Tetracycline resistance was conferred by the presence of *tet* gene determinants, where *tet*K was found in 12 (42.86%) and *tet*L was detected in 5 (17.86%) isolates, respectively (Figure 4). However, the *erm*C and *tet*M genes were not detected from any isolate (Table 4).

**Table 4.** Detection rate of antimicrobial resistance and virulence genes among *S. pseudintermedius* carried by clinically healthy and diseased cats.

Organism	Category	Target genes	Number of isolates	Detection rate (%)
*S. pseudintermedius* (28)	Resistant genes	*mec*A	5	17.86
		*erm*A	7	25.0
		*erm*B	3	10.71
		*erm*C	0	0
		*tet*K	12	42.86
		*tet*L	5	17.86
		*tet*M	0	0
	Virulence genes	*sea*	9	32.14
		*seb*	3	10.71
		*sec*	0	0
		*pvl*	4	14.29
		*eta*	2	7.14
		*etb*	0	0
		*tst*	7	25.0

### Distribution of virulence genes

**Figure 5.**
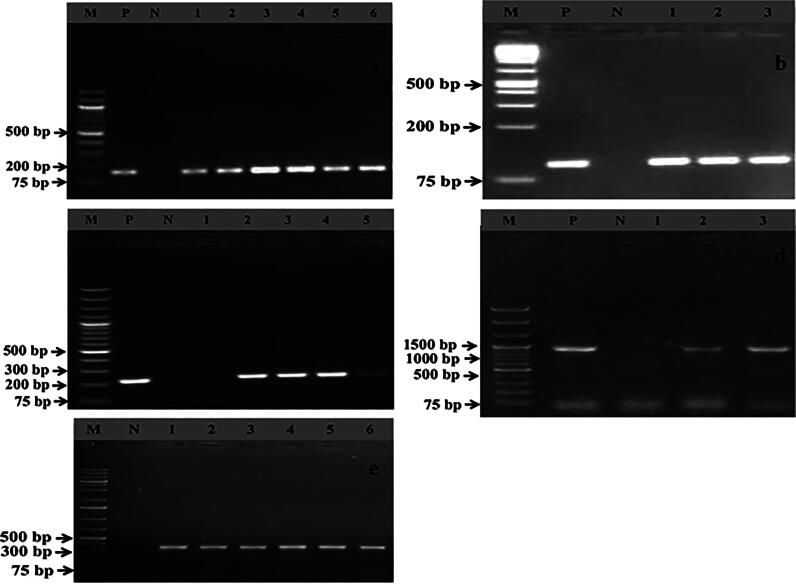
Gel electrophoresis images (5a–5e) depict the amplified PCR products of different virulence genes, namely *sea* (102 bp), *seb* (164 bp), *pvl* (255 bp), *eta* (1155 bp), and *tst* (326 bp), respectively. In all gel electrophoresis processes, a 1 kb plus DNA marker was used to confirm the amplification of a specific virulence gene. Additionally, lanes M, N, and P represent the DNA markers, negative control, and positive control, respectively.

All isolates were screened for the detection of virulence genes (Figures 2 and 5). In total, 12 (42.86%) isolates were found positive for enterotoxin genes. Among them, the *sea* gene was harbored in 9 (32.14%) isolates, followed by *seb* in 3 (10.71%) isolates (Figure 5). The toxic shock syndrome toxin-1 (*tst-*1) gene was detected in 7 (25%) isolates whereas, the Panton-Valentine leukocidin (*pvl*) was found in 4 (14.29%) isolates. Only two (7.14%) isolates carried exfoliative toxin gene (*eta*) (Figure 5). None of the isolates had the *sec* and *etb* genes (Table 4).

## Discussion

In the present cross-sectional study, we investigated the occurrence of coagulase-positive *S. pseudintermedius* in cats and assessed their antimicrobial resistance patterns, associated resistance and virulence determinants. Since the prevalence of MRSA and MRSP has been reported in dogs (Rana et al. 2022a), however, the occurrence of *S. pseudintermedius* and related microbiological studies has seemingly never been reported in cats in Bangladesh.

The phenotypic identification of *S. pseudintermedius* was based on the observation of colony morphology on blood agar and MSA plates. On blood agar, colonies had a diameter of 1-2 mm, were smooth, slightly convex, opaque to white, and lacked pigmentation (Markey et al. 2013). The colonies of *S. pseudintermedius* typically exhibit haemolytic properties on blood agar. The phenotypic identification sometimes creates bewilderment to others *Staphylococcus* species on blood agar, especially the bacteria that produce opaque to white color colonies (Markey et al. 2013). *S. pseudintermedius* produces both alpha- and beta-haemolysin on blood agar, where the alpha-haemolysin produces a zone of complete haemolysis immediately surrounding the colony, while the beta-haemolysin generates a zone of partial or incomplete haemolysis. This phenomenon was commonly known as double haemolysis (Quinn et al. 2011). On MSA, *S. pseudintermedius* ­produced opaque, shiny, whitish colonies with the surrounding medium turning yellow. While *S. pseudintermedius* generally exhibited coagulase positivity and the majority of isolates fermented mannitol salt, leading to acid production and a consequent changed in the phenol red indicator from pink to yellow, there were instances of certain strains of *S. pseudintermedius* that demonstrated weak mannitol fermentation. In such cases, these strains didn’t generate sufficient acid to induce the characteristic yellow color changed in the media (Kadhim and Abdullah 2022). In contrast, coagulase-negative staphylococci lack the ability to ferment mannitol, resulting in the pink coloration of the surrounding medium (De la Maza et al. 2020; Kadhim and Abdullah 2022).

The present study revealed that the prevalence of *S. pseudintermedius* in cats was 12.1%. The prevalence of *S. pseudintermedius* in this study was somewhat greater when compared to prior findings. According to Ma et al. (2020), the prevalence of *S. pseudintermedius* in cats was 6.5% in Australia and Bierowiec et al. (2021) reported 2.49% in healthy cats and 7.61% in sick cats in Poland. The occurrence of *S. pseudintermedius* was relatively lower in felines when compared to canines (Ma et al. 2020). The prior literature reported the prevalence of *S. pseudintermedius* in cats to range from 2.49% to 8.8%, on the contrary in dogs, the prevalence has been reported to have a range of 45 to 90% (Ma et al. 2020; Bierowiec et al. 2021; Rana et al. 2022a). Therefore, it may possible that *S. pseudintermedius* didn’t constitute a native microbiota of cats (Smith et al. 2020) and study reported that *S. pseudintermedius* was usually obtained from cats cohabiting with dogs (Ma et al. 2020). However, a wide range of factors, such as breed, age, sex, management approaches, clinical conditions, geographical location, contact with pet dogs and sampling size can influence the prevalence of *S. pseudintermedius* in cats.

As a cutaneous commensal *S. pseudintermedius* was commonly isolated from the mouth, nares, pharynx, forehead, groin, and anus of asymptomatic dogs but less frequently from cats (Garbacz et al. 2013; Nocera et al. 2023). In our investigation, the isolation rates of *S. pseudintermedius* from the perineum and oral cavity were 10.78% and 8.82%, respectively. The carriage of *S. pseudintermedius* in different body sites of cats has been reported to vary significantly. For instance, Lilenbaum et al. (1998) found that 26.5% of isolates obtained from skin scrapings of healthy adult cats were positive for *S. pseudintermedius* in Brazil. Similarly, Gandolfi-Decristophoris et al. (2013) reported a prevalence of 2.7% for isolates obtained from the nares and external ear canals of healthy pet cats in Switzerland. The isolation of *S. pseudintermedius* appears to be more prevalent in the perineum compared to the anterior nares or oropharynx (Moses et al. 2023). These body sites have been identified as the most often colonized sites for *S. pseudintermedius* in dogs that have also been true for cats as reported in various investigations (Garbacz et al. 2013; Ma et al. 2020).

The occurrence of *S. pseudintermedius* in dermatitis and otitis cases was found only in 26.08% and 33.33% cats, respectively. Bierowiec et al. (2021) also reported a lower incidence (7.61%) of *S. pseudintermedius* in sick cats in Poland. *S. pseudintermedius* was infrequently isolated from cutaneous lesions in cats compared to dogs. Nevertheless, few early reports have identified the organism from cats with respiratory tract infections, urogenital infections, conjunctivitis, dermatitis, otitis, and wounds (Lehner et al. 2014; Bierowiec et al. 2019). Moreover, due to frequent colonization in different anatomical sites, it has the ability to produce clinical conditions or aggravate any secondary conditions like immunosuppression, post-surgical contamination, suppuration, and others in pet animals.

The study focused that *S. pseudintermedius* exhibited a significant spectrum of resistance to the 12 antimicrobial drugs. The existence of a varied antimicrobial resistance profile in *S. pseudintermedius* has been documented in several prior research (Gómez-Sanz et al. 2011; Bierowiec et al. 2021; Rana et al. 2022a; Viegas et al. 2022; Wang et al. 2022; Morais et al. 2023). A total of 89.28% of isolates showed multi-drug resistance which was higher than the previous findings of Bierowiec et al. (2021) who reported MDR in 50% of healthy cats and 38.46% of sick cats.

The majority of the *S. pseudintermedius* isolates examined in this study exhibited resistance to penicillin (96.42%), streptomycin (85.71%), and erythromycin (78.57%). Resistance to amoxicillin/clavulanic acid and ceftriaxone were seen in 60.71% and 64.28% of the isolates, respectively. The high resistance frequency of *S. pseudintermedius* against erythromycin was described in multiple earlier researches (Bierowiec et al. 2021; Viegas et al. 2022; Wang et al. 2022). The possible cause of this occurrence could be attributed to the frequent irrational administration of identical antimicrobial agents for treating infections or the improper choice of antibiotics for the treatment of various clinical conditions. Furthermore, the utilization of antimicrobial agents belonging to analogous groups, which exhibit comparable mechanisms of action against a specific organism, may potentially promote the emergence of MDR (Rana et al. 2022a). The susceptibility of gentamicin was found to be 92.86%, indicating a high level of effectiveness. This was closely followed by imipenem, with a susceptibility rate of 89.28%. The high susceptibility for gentamicin and imipenem could potentially be attributed to the comparatively lower rate of administration of these antibiotics in pet animal practices in the context of Bangladesh.

The study revealed an overall prevalence of MRSP was 17.86%. However, the lower incidence of colonization by *S. pseudintermedius*, including MRSP was reported in previous different studies (Couto et al. 2011; Nienhoff et al. 2011; Lehner et al. 2014). The presence of MRSP in cats suggests that the infections might be originate from hospital or clinic settings or could be direct or cross transmission from dogs (Lehner et al. 2014). MRSP strain shows resistance to all β-lactam antibiotics and *mec*A gene-encoding bacterium has the ability to produce β-lactamase enzymes, which are the primary cause of β-lactam antimicrobial drug resistance (Rana et al. 2020b, Bush and Bradford 2020). Repeated use of β-lactam antibiotics, acquisition of resistant genes from other organisms, and chromosomal mutation might be a reason for the emergence of MRSP in cats. However, MRSP also associated with clinical infection including dermatitis, pyoderma, otitis and suppurative condition in companion animals and create a significant health burden as well as challenge for clinical management. MRSP has been reported as a significant pathogen in the field of veterinary medicine particularly for pet animals like MRSA in humans and food animals (Moses et al. 2023, Rana et al. 2022b, Rana et al. 2020b). The presence of MRSP in pet animals is leading to great concern over the treatment and control of infections and majority of cases infections often exhibit a recurring pattern, resulting in animals undergoing multiple cycles of antibiotic therapy (Morris et al. 2017).

Both phenotypic and genotypic evidence of resistance to erythromycin and tetracycline were determined in this study. The study revealed that 25% of isolates harbored the *erm*A and 10.71% *erm*B gene, which confirmed the genetic determinant of erythromycin resistance. The presence of tetracycline-resistant genes, specifically *tet*K and *tet*L were observed in 42.86% and 17.86% of isolates, respectively. These findings are closely similar to the previous studies where it reported *erm*B, *tet*K and *tet*L were frequently isolated from pet animals particularly in dogs and cats (Bierowiec et al. 2021; Morais et al. 2023). However, the evolution of different erythromycin and tetracycline genes indicates the emergence of MDR isolates (Rana et al. 2022b). Selecting the suitable antimicrobials for treatment of companion animals is becoming increasingly complex due to rapid evolution of MDR pathogen. Moreover, the zoonotic potential of *S. pseudintermedius* is well-documented. Thus, it is highly recommended to undertake continuous monitoring of phenotypic and genotypic antimicrobial resistance profiles before choosing therapeutic antimicrobials for animal treatment (Bierowiec et al. 2021; Rana et al. 2022b).

In present study, we identified diverse virulence genes of *S. pseudintermedius* that act as a triggering factor for the genesis of clinical condition in animals. The enterotoxin gene like *sea* was identified in 32.14% of the isolates, followed by the *seb* gene in 10.71% of isolates. The *tst* gene was identified in 25% of isolates, while the *pvl* gene was identified in 14.29% of isolates. Strain of *S. pseudintermedius* isolates were found to contain *sea*, *seb*, *sec*, *see*, and *tst-*1 virulence genes as documented in previous different studies (Rahmdel et al. 2018; Meroni et al. 2019). The Panton-Valentine leucocidin (*pvl*) gene exhibits a high degree of similarity to the leukotoxin *Luk*-I, which demonstrates potent leucotoxic effects on different types of polymorphonuclear cells of host body (González-Martín et al. 2020). The pathogenicity of *S. pseudintermedius* is associated with the existence of many virulence genes, structural components and host factors, where majority are closely resemble to *S. aureus*. The virulence genes and coagulase enzyme component described in this study were involved in various stages of bacterial growth, including colonization, infection, and dissemination.

However, there are some limitations to the current study. We sampled the cats that were registered with the hospital without estimating the statistical sample size. We have not identified the specific risk factors associated with the occurrence of *S. pseudintermedius* in cats. Due to resource constraints, we were unable to conduct extensive genotyping on the isolates. The details molecular insights of *S. pseudintermedius* resistant and virulence genes in cats would be extremely beneficial for further understanding the characteristics of each isolate. Future studies should overcome these limitations.

## Conclusion

The carriage of *S. pseudintermedius* in cats was 12.01%. Alarming findings are the significant number of the isolates exhibited MDR and a substantial proportion of isolates harbored the *mec*A gene, which confirmed the methicillin resistance. In addition, the presence of erythromycin resistant genes *erm*A (25%), *erm*B (10.71%), and tetracycline resistant genes *tet*K (42.86%), *tet*L (17.86%), along with several virulence genes including *sea* (32.14%), *seb* (10.71%), *tst*-1(25%), and *pvl* (14.29%), indicates that MRSP poses a clinical burden not just to cat populations but also create potential zoonotic risk to pet owners as well as intertwined veterinarian.

## Data Availability

The data represents the findings of this study are available within the article.
